# Alate susceptibility in ants

**DOI:** 10.1002/ece3.1291

**Published:** 2014-10-20

**Authors:** Eddie K H Ho, Megan E Frederickson

**Affiliations:** 1 Department of Ecology and Evolutionary Biology, University of Toronto25 Willcocks Street, Toronto, ON, M5S 3B2, Canada; 2 Department of Ecology and Evolutionary Biology, University of Toronto25 Harbord Street, Toronto, ON, M5S 3G5, Canada

**Keywords:** Alate susceptibility, ants, *Beauveria bassiana*, division of labor among castes, haploid or male susceptibility, individual and colony immunity, levels of selection

## Abstract

Pathogens are predicted to pose a particular threat to eusocial insects because infections can spread rapidly in colonies with high densities of closely related individuals. In ants, there are two major castes: workers and reproductives. Sterile workers receive no direct benefit from investing in immunity, but can gain indirect fitness benefits if their immunity aids the survival of their fertile siblings. Virgin reproductives (alates), on the other hand, may be able to increase their investment in reproduction, rather than in immunity, because of the protection they receive from workers. Thus, we expect colonies to have highly immune workers, but relatively more susceptible alates. We examined the survival of workers, gynes, and males of nine ant species collected in Peru and Canada when exposed to the entomopathogenic fungus *Beauveria bassiana*. For the seven species in which treatment with *B. bassiana* increased ant mortality relative to controls, we found workers were significantly less susceptible compared with both alate sexes. Female and male alates did not differ significantly in their immunocompetence. Our results suggest that, as with other nonreproductive tasks in ant colonies like foraging and nest maintenance, workers have primary responsibility for colony immunity, allowing alates to specialize on reproduction. We highlight the importance of colony-level selection on individual immunity in ants and other eusocial organisms.

## Introduction

All organisms are infected by parasites and pathogens. As a result, a role for disease has been invoked in diverse areas of evolutionary biology, including the evolution of sex (Hamilton 1980), the maintenance of genetic variation (King et al. 2011), host defense dynamics (Débarre et al. 2012), and social evolution (O'Donnell and Beshers 2004). The negative effect of parasites on their hosts creates strong selection for host immunity, and a large literature has investigated how host immunocompetence varies with factors such as sex (Zuk and McKean 1996; May 2007; Nunn et al. 2009), age (Laughton et al.^2011^; McNamara et al. 2012), body size (Moore and Wilson 2002; Hughes et al. 2010), genetic diversity (O'Donnell and Beshers 2004; Ugelvig et al. 2010), and life history (Stoehr and Kokko 2006; Schmidt et al. 2011). Eusocial insects provide a particularly interesting model for studying immunity because of the large variation in life span, physiology, and behavior among species and also among castes within a species.

One of the hallmarks of eusociality is a reproductive division of labor. In ants, queens lay eggs that develop into workers, gynes (i.e. virgin queens) or males. (Mostly) sterile workers perform tasks such as foraging, defense, and nest maintenance, whereas gynes and males are the reproductive members of the colony that eventually emerge from the nest, mate, and establish the next generation. Gynes and males are collectively called alates because they develop wings in most species, although queens shed their wings soon after mating (Hölldobler and Wilson 1990). Because eusociality results in highly related individuals living in dense aggregations with frequent physical contact, colonies are expected to be highly susceptible to infection (Steinhaus 1958). Thus, ant colonies likely experience strong selection for immunity, but how does selection act on immunity-related traits at the individual and colony levels in ants?

To resist pathogens and parasites, ants depend on both individual and social immunity. In ant colonies, social behaviors such as allogrooming and undertaking help prevent the introduction and spread of disease (Traniello et al. 2002; Cremer et al. 2007; Reber et al. 2011). At the individual level, ants perform hygienic behaviors, such as self-grooming, make immune proteins such as phenoloxidases (Wilson-Rich et al. 2009b; González-Santoyo and Córdoba-Aguilar 2012) and also secrete antibiotic compounds (Graystock and Hughes 2011). A structure, that is, unique to ants, the metapleural gland, produces secretions for antisepsis and hygiene (Hölldobler and Engel-Siegel 1984; Yek and Mueller 2011). Immunological defenses, however, are not distributed uniformly among colony members. For example, workers likely enact most social immunity behaviors and in many species, males lack metapleural glands (Hölldobler and Engel-Siegel 1984). This variation in immunity among castes may result from selection pressures acting on, and potentially trading-off between, individual ants and whole ant colonies (Cremer and Sixt 2009).

Life history trade-offs often contribute to differential immunity between the sexes (Rolff 2002; Stoehr and Kokko 2006). Males and females typically differ in how they maximize reproductive output, an idea referred to as Bateman's principle (Bateman 1948; Trivers 1972). Males invest relatively little in producing offspring and gain fitness largely through increased mating success. Females supply much of the reproductive effort and are limited in the number of offspring they can produce; they gain fitness largely through longevity. Rolff (2002) examined Bateman's principle with regard to immunity and concluded that females should invest more in immunity than males. In general, longevity correlates with infection risk and thus females should invest in immunity to prolong life span. The fitness of males is more limited by their ability to fertilize females and thus males should invest less in immunity and more in traits that benefit mating success. Life history differences lead to the prediction that females should be more immunocompetent than males.

The life histories assumed by Bateman's principle apply to many ant species (Hölldobler and Wilson 1990). Gynes live much longer than males, especially if they go on to successfully found colonies. They must mate, establish new colonies and survive to produce the next generation of alates, all of which increase the risk of pathogen exposure. Males, on the other hand, spend the majority of their lives within natal nests, only venturing out to mate and dying shortly thereafter (Hölldobler and Wilson 1990; Boomsma et al. 2005). Thus, males are only briefly exposed to pathogens outside the nest. Furthermore, even if they are infected, males may still be able to mate before they succumb to infection (Stoehr and Kokko 2006). Therefore, infection is likely to have a much larger negative impact on the reproductive output of gynes than males. We would predict that male ants invest less in immunity than gynes and, as a result, be more susceptible to pathogens (Wilson-Rich et al. 2009b).

Rolff (2002) did not examine how selection might act on males and females in colonies of ants or other eusocial insects. Because of the interdependency of ant castes for survival and reproduction, the colony as a whole is the primary unit of selection (Cremer and Sixt 2009). Examining any individual caste without considering its role in the colony does not provide a full picture for the evolution of immunity-related traits. Thus, although maintaining a competent immune system can come at a considerable metabolic cost to individuals, often reducing investment in other functions such as growth or reproduction (Lochmiller and Deerenberg 2000; Poulsen et al. 2002; McNamara et al. 2012), this individual-level trade-off is insufficient to explain variation in immunity among ant castes. For example, workers are typically sterile, so investing resources into immunity does not come at a cost to their reproduction, nor does the increased survival provided by immunity aid in their reproductive success. Instead, if highly immune workers carry few pathogens into the confines of their nest or limit the spread of pathogens that do reach the nest, they gain inclusive fitness benefits by preventing infection of their fertile siblings. Alates may take advantage of the protection offered by highly immune workers by investing more in growth, reproduction, and energy storage but relatively less in immunity. In short, natural selection may favor colonies with workers that make costly immunological defenses if this boosts alates' reproductive success, leading us to predict that workers should be more immunocompetent than alates. Life history differences may still act to select for increased immunocompetence in gynes relative to males.

The genetic system of hymenoptera provides an alternative explanation for increased male susceptibility. All Hymenoptera are haplodiploid, such that males are haploid and females are diploid. O'Donnell and Beshers (2004) predicted that males should be more susceptible to infection because of a reduction in the number of loci conferring immunity (assuming additivity) or decreased allelic diversity at these loci. In ants, previous studies comparing males to either workers or queens have found lower immunocompetence in males (Vainio et al. 2004; Baer et al. 2005). Similarly, in bees, males have either a lower or an equal immune response as workers (Baer and Schmid-Hempel 2006; Ruiz-gonzález and Brown 2006; Laughton et al. 2011). However, ploidy and life history differences are predicted to have similar effects on the relative immunity of male and female hymenopterans, making it difficult to separate these factors. Furthermore, comparing males to workers (but not gynes) may confound sex and caste differences in immunity.

From considerations of life history and colony-level selection, we expect alate susceptibility, with workers being more immunocompetent than alates, and gynes more immunocompetent than males. Under haploid susceptibility, however, we expect both female castes to be equally immunocompetent and more immunocompetent than males. Although these hypotheses are nonmutually exclusive, we must examine both reproductive sexes and workers to fully understand selective pressures acting on immunity within colonies. Some previous studies have examined two of these three castes; males were less immunocompetent than either workers or queens, whereas comparisons between workers and gynes gave mixed results (Vainio et al. 2004; Baer et al. 2005; Hughes and Boomsma 2006, Hughes et al. 2010; Sorvari et al. 2008; Vitikainen and Sundström 2011). One of the few studies to examine immunity in all three ants castes recorded the presence of parasitic Strepsiptera and found some evidence for male or alate susceptibility (Hughes et al. 2003; see also Kathirithamby and Johnston 1992; Kathirithamby and Hughes 2002). However, observational studies do not account for deaths that occurred before observation, nor for the abundance of each caste within a colony, which may affect how often parasites come in contact with hosts. Therefore, experimental studies are better suited for assessing individual immunity.

This study examines immunity in nine species of ants collected from Peru and Canada. We tested the immunocompetence of males, gynes, and workers by inoculating them with a control solution or a suspension containing the generalist entomopathogenic fungus, *Beauveria bassiana,* housing them individually in test tubes, and recording their survival; this method precludes any traits involved in social immunity. Our study is the first to examine how immunocompetence varies among castes in a variety of ant species.

## Methods

### Study sites and species

In Peru, we collected ants from several habitats at the Centro de Investigación y Capacitación Rio Los Amigos (“CICRA,” 12°34′S, 70°05′W; elevation ∼270 m), which is a biological station located at the confluence of the Madre de Dios and Los Amigos rivers. Surrounding the station is the Los Amigos conservation concession, which comprises 146,000 ha of mostly primary tropical rainforest on a mixture of upland terraces and floodplains. Annual rainfall at Los Amigos is between 2700 and 3000 mm, with more than 80% of the precipitation falling during the October–April wet season (Pitman 2008). Mean monthly temperatures range from 21 to 26°C and humidity averages 87% (Pitman 2008). In Canada, we collected ants from the Koffler Scientific Reserve at Jokers Hill (“KSR,” 44°02′N, 79°32′W; elevation ∼300 m) and elsewhere in the greater Toronto area. Annual precipitation in southern Ontario averages 790 mm and mean monthly temperatures range from −6°C in January to 21°C in July (Environment Canada, Canadian Climate Normals 1971–2000).

Excluding callows, we collected live workers (minors only in polymorphic species), gynes, and virgin males of nine ant species (Table 1) for use in survival assays. Ants were brought back to the laboratory and kept in plastic containers, fed standard artificial diet (Bhatkar and Whitcomb 1970), and given water via a damp piece of cotton. All ants were assayed within 24 h of collection. In Peru, we hand-collected arboreal ants from bamboo culms (*Camponotus mirabilis* and *C. longipilis*) and from the hollow stems of myrmecophytic *Cordia nodosa* trees (*Allomerus octoarticulatus* and an unidentified and possibly undescribed species of *Azteca*). We also collected colonies of *Odontomachus bauri* from decaying wood or soil. In Canada, we collected colonies of four species of ground-dwelling ants: *Aphaenogaster* cf. *rudis* and *Lasius* cf. *nearcticus* from decaying wood and *Myrmica rubra* and *Brachymyrmex depilis* from soil. We chose these ant species because they had multiple colonies producing alates at the time of our study; they are not a random sample from across the ant phylogeny, but we have no reason to think they differ systematically from other ants with respect to immunity-related traits. All colonies contained workers and at least one of the alate sexes (sometimes both). Because split sex ratios occur in many ant species, some colonies may contain only gynes or males at a given time (Meunier et al. 2008).

**Table 1:** Number of individual ants within each treatment and their average body length for each caste and species examined

				Ants in fungus, control treatments			Average body length, mm (replicates)		
Subfamily	Species	Nest type	Colonies	Workers	Gynes	Males	Workers	Gynes	Males
*Peru*									
Myrmicinae	*Allomerus octoarticulatus*	Arboreal	6	36, 35	10, 10	22, 22	1.72 (12)	5.46 (7)	4.79 (6)
Dolichoderinae	*Azteca* sp.	Arboreal	2	11, 12	0, 0	9, 9	2.42 (15)		1.9 (16)
Formicinae	*Camponotus mirabilis*	Arboreal	6	27, 27	9, 7	23, 23	5.92 (12)	12.25 (8)	5.99 (4)
Formicinae	*Camponotus longipilis*	Arboreal	4	14, 13	8, 8	10, 10	7.42 (7)	10.54 (1)	6.76 (3)
Ponerinae	*Odontomachus bauri*	Soil	4	26, 26	13, 12	4, 4	5.53 (17)	6.37 (4)	4.16 (6)
*Canada*									
Myrmicinae	*Aphaenogaster* cf. *rudis*	Soil	7	43, 39	37, 37	18, 16	3.29 (15)	5.17 (5)	3.54 (12)
Formicinae	*Brachymyrmex depilis*	Soil	2	12, 12	11, 9	6, 6	1.05 (5)	2.99 (5)	1.47 (5)
Formicinae	*Lasius* cf. *nearcticus*	Soil	2	12, 12	10, 10	12, 12	2.61 (12)	4.95 (5)	2.88 (21)
Myrmicinae	Myrmica rubra*	Soil	2	30, 30	0, 0	26, 27	3.36 (15)		3.52 (17)

* Exotic species.

### Survival assays

We exposed ants to the generalist entomopathogenic fungus, *Beauveria bassiana*, which infects over 200 species of arthropods and has been used in other studies of ant immunity (Feng et al. 1994; Diehl and Junqueira 2001; Schmidt et al. 2011). *Beauveria bassiana* is not actively used as an insecticide at our field sites (CICRA: M. Frederickson, pers. obs.; KSR: A. Weis, pers. comm.). We extracted conidia from the commercial insecticide Botanigard ES (strain GHA) by first growing a suspension on 6.5% sabouraud dextrose agar plates in a darkened environment. To avoid contamination by other chemicals in Botanigard ES, conidia from these initial plates were not used directly. Instead, we collected these conidia and grew them on new plates; conidia arising from these secondary plates were used for survival assays. We suspended the conidia in a 0.05% solution of the surfactant Triton X-100 [Sigma-Aldrich, Oakville, Ontario, Canada]. We counted conidia densities using a haemocytometer and diluted the suspension to a concentration of 1 × 10^7^ conidia/mL. This was procedure was performed daily to ensure a fresh supply of conidia. Conidia suspensions were checked to be viable by plating them on 6.5% sabouraud dextrose agar plates. In the fungal and control treatments, respectively, we placed 0.5 *μ*L of the conidia suspension or the same amount of a 0.05% solution of the surfactant only on ant thoraces.

The number of workers and alates collected from each colony varied depending on the quantity available. We used approximately equal numbers of each caste (i.e. workers, males, gynes) for the fungal and control treatments (Table 1). For two species, *Azteca* sp. and *M. rubra*, we were unable to collect gynes (Table 1). All individuals within a colony were exposed to the same fungal suspension and the same suspension was used for colonies and species collected on the same date. We placed each fungus-treated or control ant in a 50-mL falcon tube and kept the tubes at ambient temperatures (in Peru, ∼L12:D12 light cycle, ∼18–33°C) or in environmental chambers (in Canada, L14:D10 light cycle, 15–25°C). Ants were fed a standard artificial diet (Bhatkar and Whitcomb 1970) and provided with water via a damp piece of cotton. We monitored ants every day for 14 days, recording the day of death if it occurred in this period. After an ant died, it was removed from its falcon tube and placed into a 2-mL microcentrifuge tube with a small piece of damp cotton to keep the environment moist. We then monitored the deceased ants for fungal growth daily for 7 days. Over 95% of ants that died in the fungal treatment and just 1% of ants that died in the control treatment had *B. bassiana* hyphae growing out of their corpses within 7 days. This suggests that the differences in survival between the treatments were due to *B. bassiana* exposure. In total, we monitored the survival of 445 fungus-treated and 434 control ants.

### Body size

We measured ant body size on a different set of individuals from the ants used in the survival assays, but all were collected at the same time and from the same sites. Under a Leica M205 dissecting microscope with a digital micrometer, we measured the maximum length of the head, mesosoma, petiole, and gaster of each ant, and then summed these to get a measure of body length. The number of individuals per caste per species varied from 1 to 20 (Table 1). Although these measures are not from the individuals used in the survival assays, we have no reason to expect biases in body size among the ants used in the assays and the ants used for size measurements.

### Statistical analysis

We assessed variation among workers, gynes, and males in susceptibility to *B. bassiana* only for species in which the fungal treatment significantly affected ant mortality. We tested whether the fungal treatment significantly affected the mortality of all nine species independently using a Cox proportional hazard model, with caste, treatment, and colony as main effects. We found that the fungal treatment had no significant effect on *Azteca* sp. and *L. nearcticus* mortality. We checked this by performing a likelihood ratio test between the full model and a model with just caste and colony (fungal treatment removed) for these two species. The full model did not provide a better fit for the data in *Azteca sp*. (*P* = 0.1564) or *L. nearcticus* (*P* = 0.4039). This lack of response is largely attributable to the high baseline mortality of males in both species (3.33 days in *Azteca* sp. and 4 days in *L. nearcticus*, respectively), which greatly constrains the potential effect size of the fungal treatment. Our study would be unable to adequately assess variation in susceptibility among castes for either species, and thus they were removed from further analyses.

For the remaining seven species (*C. mirabilis*, *C. longipilis*, *A. octoarticulatus*, *O. bauri*, *M. rubra*, *A. rudis,* and *B. depilis*), we used survival analysis to analyze our data, with the Cox proportional hazards model censored at 14 days. Treatment, caste, and species were included as main effects; site of origin (Peru or Canada), colony of origin, and type of nest (arboreal or ground) were included as random factors but were not significant predictors of survival and were removed from the final model. The regression coefficients for all three-way interactions (treatment × caste × species) were nonsignificant in the full model, so we included only the two-way interactions in the final model (*i.e.,* treatment × caste, treatment × species, and caste × species). A log-likelihood ratio test indicated that the full model, including three-way interactions, did not improve model fit, compared with a model with only two-way interaction terms (*P* = 0.122). We also created Kaplan–Meier curves for each caste with treatment as the main factor. Unlike our main statistical model, it pools all species together, but nonetheless provides a useful visualization of the results.

We calculated hazard ratios (HR) from the coefficients in the Cox regression. The hazard ratio represents the probability of one group dying relative to another group at any point in time. Values above one indicate an elevated risk of dying, while values below one indicate the opposite. We only use regression coefficients significant at the ≤0.05 level. By calculating the HR of gynes or males relative to workers in the control treatment, we can determine if baseline mortality differed among castes. However, we are most interested in whether mortality due to the fungal treatment differed among castes. To examine this, we calculated the treatment-HR (HR_trt_), which is the HR of the fungus-treated group relative to the control group for each caste and species individually; this takes into account any differences in baseline mortality between castes and species. We can then make comparison among HR_trt_ values to determine whether they differed among castes within a species (Altman and Bland 2003). Significant differences in HR_trt_ indicate that the fungus treatment had different effects on castes, suggesting differences in caste immunity.

Lastly, we investigated the relationship between body size and immunity. As mentioned previously, body size was measured from a different set of ants collected from the same colonies as those used for the survival assays and thus cannot be incorporated directly into the survival analysis. To investigate if body size is a predictor of immunity, we used Ln (HR_trt_) as a proxy for immunocompetence. We fit an ANCOVA with average body size, caste, and species as main effects to determine whether these are significant predictors of Ln (HR_trt_). Due to limitations in statistical power, we could not create a full model that accounts for all two-way and three-way interactions. Instead, we fit the data to two separate models. *Model 1*: main effects and interaction effects of body size and caste with species as a cofactor. *Model 2*: main effects and interaction effects of body size and species with caste as a cofactor. However, backwards elimination removed interaction effects from both *Model 1* and *Model* 2. Log-likelihood ratio tests favored a reduced model without interaction effects in body size x caste (*Model 1*; *P* = 0.475) and in body size x species (*Model 2*; *P* = 0.826). We report results from this reduced model. We also report Bonferroni-corrected *P*-values to account for the use of HR_trt_ in multiple tests.

For all analyses, we utilized the downloadable packages, *Survival* and *ggplot2*, in the statistical software *R* (R Development Core Team RFFSC 2013).

## Results

**Figure 1:**
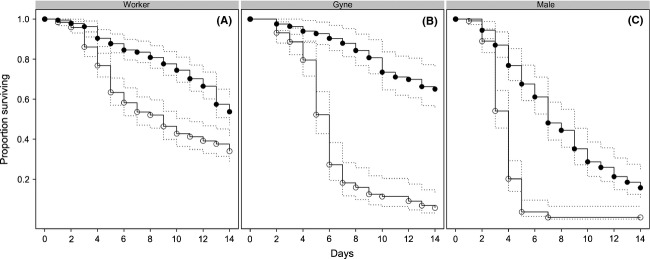
Kaplan–Meier curves (±95% confidence intervals) showing the proportion of control (closed circles) and fungus-treated (open circles) ants surviving over 14 days. Data from all species, excluding *Azteca* sp. and *Lasius* cf. *nearcticus*, are combined and plots are separated by caste: (A) worker, (B) gyne, and (C) male. The figure illustrates the general trends in the results.

For seven of the nine species we tested, ants survived significantly longer in the control than in the fungus treatment. In these seven species, alates were more susceptible to the fungus treatment than workers. Kaplan–Meier plots provide a useful visualization of the different survival rates among castes under the control and fungus treatments (Fig. 1), although these plots incorrectly pool all species together. The results of the final Cox survival model, which includes species and its interactions with treatment and caste as factors, show that the fungus treatment significantly increased mortality (*β* = 0.516, *P* = 0.026) and, more importantly, the effect of the fungus treatment differed among castes, as indicated by a significant interaction between fungus treatment and being a gyne (*β* = 0.941, *P* < 0.001) or a male (*β* = 1.139, *P* < 0.001). Select regression coefficients from the Cox survival analysis are in Table 2; the full results, including all nonsignificant coefficients, are in Table S1. Positive (negative) coefficients indicate an increase (reduction) in mortality.

**Table 2:** Select regression coefficients from the Cox proportional hazards analysis (full results in Table S1)

	Regression coefficient	Standard error	Lower 95% CI	Upper 95% CI	*P*-value
Fungus	0.516	0.232	0.061	0.970	0.026
Worker	0.0000				
Gyne	−0.871	0.391	−1.637	−0.104	0.026
Male	0.979	0.240	0.508	1.450	<0.001
Fungus × Gyne	0.941	0.275	0.403	1.479	<0.001
Fungus × Male	1.139	0.219	0.711	1.567	<0.001

**Figure 2:**
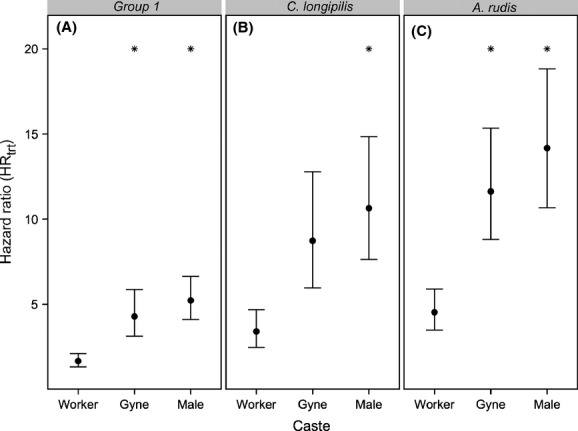
Hazard ratios (± standard error) of fungus-treated and control ants in each caste (HR_trt_) separated into three groups: (A) *Allomerus octoarticulatus*, *Brachymyrmex depilis, Camponotus mirabilis*, *Myrmica rubra,* and *Odontomachus bauri* (Group 1), (B) *Camponotus longipilis* and (C) *Aphaenogaster* cf. *rudis*. Asterisks indicate significant differences in HR_trt_ between an alate caste and workers.

To investigate these effects more fully, we calculated HRs and accounted for differences among species (Table 2, Tables S1 and S2). Within the control treatment, castes and species differed significantly in their baseline mortality. Males had the highest baseline mortality, while gynes generally had similar or lower mortality relative to workers, depending on the species (Table 2, Tables S1 and S2). Although baseline mortality varied by castes and species, we focused on comparing the effects of the fungus treatment on mortality among castes within a particular species. Based on values of HR_trt_, we can sort our species into three groups. Within castes, there were no differences in HR_trt_ among *A. octoarticulatus*, *B. depilis, C. mirabilis*, *M. rubra,* and *O. bauri* (treatment × species interaction effects are nonsignificant; Table S1), indicating that the fungus treatment had similar effects on workers, gynes, and males among these five species; these species make up Group 1. HR_trt_ values for *Camponotus longipilis* and *A. rudis* were quantitatively different from Group 1 and are shown separately (Fig. 2).

In all three groups (*i.e.,* Group 1, *C. longipilis,* and *A. rudis*), the fungus treatment caused alates to suffer higher mortality than workers, as shown by the significantly larger HR_trt_ of both gynes and males relative to workers (Fig. 2). Among the species in Group 1, workers had a significantly lower HR_trt_ than gynes (*z* = 2.82, *P*_adjusted_ = 0.014) and males (*z* = 4.75, *P*_adjusted_ << 0.001), indicating that the fungus treatment affected worker mortality much less than gyne or male mortality. Similarly, *A*. *rudis* workers also had a significantly lower HR_trt_ than *A. rudis* gynes (*z* = 2.46, *P*_adjusted_ = 0.038) and males (*z* = 2.95, *P*_adjusted_ = 0.010). In *C. longipilis*, workers had a significantly lower HR_trt_ than males (*z* = 2.46, *P*_adjusted_ = 0.038), and a lower but nonsignificantly different HR_trt_ relative to gynes (*z* = 1.89, *P*_adjusted_ = 0.132). In all three groups, the large overlap in the standard errors of HR_trt_ between gynes and males indicates that the effect of the fungus treatment on ant mortality did not differ significantly between the alate sexes (Fig. 2). In short, workers tended to be less susceptible to the fungus treatment than both gynes and males, but gynes and males succumbed similarly to the fungus. Lastly, our ANCOVA model did not identify average body size as a significant predictor of Ln (HR_trt_) (*β* ≪ 0.001, *P*_adjusted_ = 1; Fig. S1).

## Discussion

This is the first study to experimentally test the immunocompetence of both reproductive sexes and workers against a pathogen in a variety of ant species. In general, we found that workers were less susceptible than either gynes or males when exposed to the generalist entomopathogenic fungus *B. bassiana*. Fungus treatment increased mortality in seven of the nine ant species we tested*,* and the effect was significantly larger for gynes and males relative to workers. Comparisons of hazard ratios show that alates of both sexes did not differ significantly in their susceptibility. Our results are consistent with a trade-off between allocating resources to immunological defenses versus reproduction, with workers and alates displaying different strategies. Alates must invest in reproduction, which reduces the resources available for immunity, while sterile workers can take on the burden of protecting the colony against disease and thereby receive inclusive fitness benefits. Our results support the hypothesis of alate susceptibility in ants and suggest that like foraging or nest maintenance, colony immunity is another task primarily accomplished by workers, thereby allowing alates to specialize on reproduction. We cannot make this conclusion for *M. rubra*, as we were unable to collect gynes, but removal of this species from the analysis did not alter our results (not shown).

An unexpected result was that gynes and males were similarly susceptible to *B. bassiana*, relative to the more immunocompetent workers. Bateman's principle predicts that males should be more susceptible to infection than females (Bateman 1948; Rolff 2002), because they should invest relatively less in immunity and more in traits that increase the likelihood of finding and copulating with mates. For example, in ants, it is suggested that the loss of metapleural glands in the males of many species may be an adaptation to reduce investment in immunity (Hölldobler and Wilson 1990; Yek and Mueller 2011). Our findings do not support classical predictions of Bateman's principle. Haploidy in males is also suggested to increase their susceptibility to pathogens (O'Donnell and Beshers 2004). Studies of ants and bees have found lower immunocompetence in males relative to one of the diploid female castes (Vainio et al. 2004; Baer et al. 2005; Baer and Schmid-Hempel 2006), and genetic diversity is important for group immunity in eusocial insects (Baer and Schmid-hempel 1999; Hughes and Boomsma 2004, 2006; Reber et al. 2008; Ugelvig et al. 2010; Schmidt et al. 2011). However, haploid susceptibility predicts that immunocompetence in all female castes should be higher than that in males—a prediction that had not been tested before our study. We found that males were only less immunocompetent relative to one of the female castes: workers, not gynes. Thus, we conclude that ploidy is not the major determinant of immunocompetence. Unfortunately, arguments based on life history and ploidy are not mutually exclusive; they both predict lower immunity in males relative to females. Yet, males and gynes were similarly susceptible to *B. bassiana*; why might this be the case?

First, it is possible there was limited scope for measuring male susceptibility because males had the highest baseline mortality. As in *Azteca* sp. and *L. nearcticus,* high baseline mortality constrains measurements of susceptibility using survival assays because the maximum effect size is limited to the difference between baseline and 100% mortality. Our ability to detect increased mortality of males relative to gynes when exposed to *B. bassiana* is thus constrained by control males having higher baseline mortality than control gynes. A different measure of immunocompetence, such as encapsulation rate, might detect a significant difference between males and gynes.

Alternatively, it is possible that distinct selective pressures act on gyne and male immunity but result in similar realized immunocompetence. For example, whereas colony-level selection may favor highly immune workers and susceptible gynes, individual-level selection acting on males may favor susceptibility because the trade-off between immunocompetence and reproductive success is stronger in males relative to females, as predicted by Bateman's principle. Analogizing immunity and foraging behavior in ant colonies may help to clarify this reasoning. In general, neither males nor gynes forage for food outside the ant nest. Males may not need to forage because they do not eat much, if at all, as adults, whereas gynes do not forage because workers forage for them. Similarly, males may invest little in immunity because they do not live long enough to need immunological defenses (Sturup et al. 2014), whereas gynes may invest little in immunity because they are protected by immunocompetent workers. Thus, we would expect male susceptibility to be adaptive even without invoking colony-level selection. However, it is likely that both individual- and colony-level selection shape male immunity as survival within their natal nest relies heavily on workers. Assessing the relative contribution of these two forces on immunity requires further study.

Ant age is also important to consider when interpreting our results, especially for the longer-lived female castes. We focused on virgin alates, but immunity likely changes as these ants mate and found colonies, for example, immunity can differ between virgin and mated queens (Schrempf et al. 2005; Baer et al. 2006; Castella et al. 2009; Galvez and Chapuisat 2014). We expect that virgin alates are only infrequently exposed to pathogens due to protection by workers, potentially selecting for low investment in individual immunity. Higher exposure to pathogens outside the colony, especially for queens founding colonies, may select for higher immunity later in life. Resources not spent on immunity at a young age may allow increased energy storage, in the form of lipid reserves (Ezmann et al. 2014), to invest in immunity at later ages. Similarly, workers may perform different tasks as they age, affecting their risk of pathogen exposure and selection on investment in immunity (Bocher et al. 2007; Robinson et al. 2009; Wilson-Rich et al. 2009a; Laughton et al. 2011). Thus, the greater susceptibility of reproductives relative to workers that we observed may not hold true throughout their ontogeny.

The alates we studied were all young enough to still be inside their natal nests. In contrast, the workers we studied were most likely a mix of younger workers performing intranidal tasks and older workers that had returned to their nests after foraging or midden work. We did not try to study ants of similar age (i.e., to compare only young workers to alates) because we were more interested in comparing the immunity of alates to that of workers that may introduce pathogens to the colony, instead of to workers of similar age; alates potentially benefit from the immunity of all of the workers (young and old) with they share a nest. Furthermore, comparisons between workers and gynes of similar age are useful only if we expect similar selection pressures for immunity at that age; this is unlikely given their highly differentiated caste roles and lifespans. To fully understand the selective pressure imposed by pathogens on ant immunity will require studying all castes at all life stages. Lacking this information, we remain conservative in restricting our hypothesis to virgin reproductives, hence the term alate susceptibility.

Body size is often found to correlate with immunocompetence (Moore and Wilson 2002), but this relationship does not appear to hold in ants (Vainio et al. 2004; Poulsen et al. 2006; Vitikainen and Sundström 2011; but see Hughes et al. 2010). Although we applied a constant dose of *B. bassiana* conidia to all ants regardless of size, there was no evidence that larger bodied ants suffered less from *B. bassiana* infection than smaller bodied ants (Fig. S1). The same dose of *B. bassiana* increased gyne mortality more than worker mortality (Fig. 2), even though workers were always smaller than gynes of the same species (Table 1). Furthermore, males were often similar in size to workers (Table 1), but they were not similarly immunocompetent (Fig. 2). Thus, we must invoke factors other than body size to explain differences in immunocompetence among castes and it is unlikely that using the same dose of inoculant for all ants, regardless of size, biased our results. Furthermore, that body size does not predict investment in immunity among ant castes is again evidence that ant immunity is influenced less by individual- than colony-level traits (i.e., we might expect a correlation with colony size, but not individual body size). It is important to note that our results do not exclude the possibility that body size within a caste, rather than among castes, is significantly associated with immunity.

Variation in immunity among castes is still poorly studied in ants and other eusocial organisms, and it is difficult to distinguish among the multiple hypotheses that make similar predictions for how costly investments in individual immunity should differ among castes. The ant colonies we studied had workers with high immunocompetence and alates that were more susceptible to the pathogen *B. bassiana*, with no consistent differences between the alate sexes. This is most easily explained as a colony-level adaptation to relieve alates, especially gynes, of the physiological burden of maintaining a robust immune system while still providing them with protection against disease. In this way, immunity is much like foraging, nest maintenance, or midden work, in that workers specialize on these tasks so that their fertile siblings may allocate more resources to reproduction. Alate susceptibility may apply more broadly than just to ants, if in other eusocial species workers also reliably defend reproductives from pathogens and thereby increase the inclusive fitness of all members of the colony.

We suggest that future studies incorporate all three major castes when examining immunity in eusocial Hymenoptera. We would have reached different conclusions if we had excluded any one caste. For example, our results would have appeared to support haploid susceptibility if we had excluded gynes. We also hope that future research will disentangle the immunological effects of different traits, such as life history, ploidy, longevity, and social behaviors, to gain a more complete understanding of the evolution of the immune system of eusocial insects. In summary, this study adds to the relatively small, but growing literature on variation in immunity among ant castes and highlights the effects that colony-level selection may have on individual immunity.

## Supporting Information

**Figure S1.** Scatterplot of average body length (mm) against Ln (Hazard ratio) for each ant caste and species. Circles, squares, and triangles represent workers, gynes, and males, respectively. Each number refers to a species: 1 – *A. octoarticulatus*, 2 – *A. rudis*, 3 – *B. depilis*, 4 – *C. longipilis*, 5 – *C. mirabilis*, 6 – *M. rubra*, 7 – *O. bauri*.

**Table S1.** All regression coefficients from the Cox proportional hazards analysis. **Table S2.** Hazard ratios by species.
