# Supplementary figures and images for: Alate susceptibility in ants
Source: Ecol Evol. 2014 Oct 20;4(22):4209–19. doi: 10.1002/ece3.1291 (PMC4267860; doi:10.1002/ece3.1291)

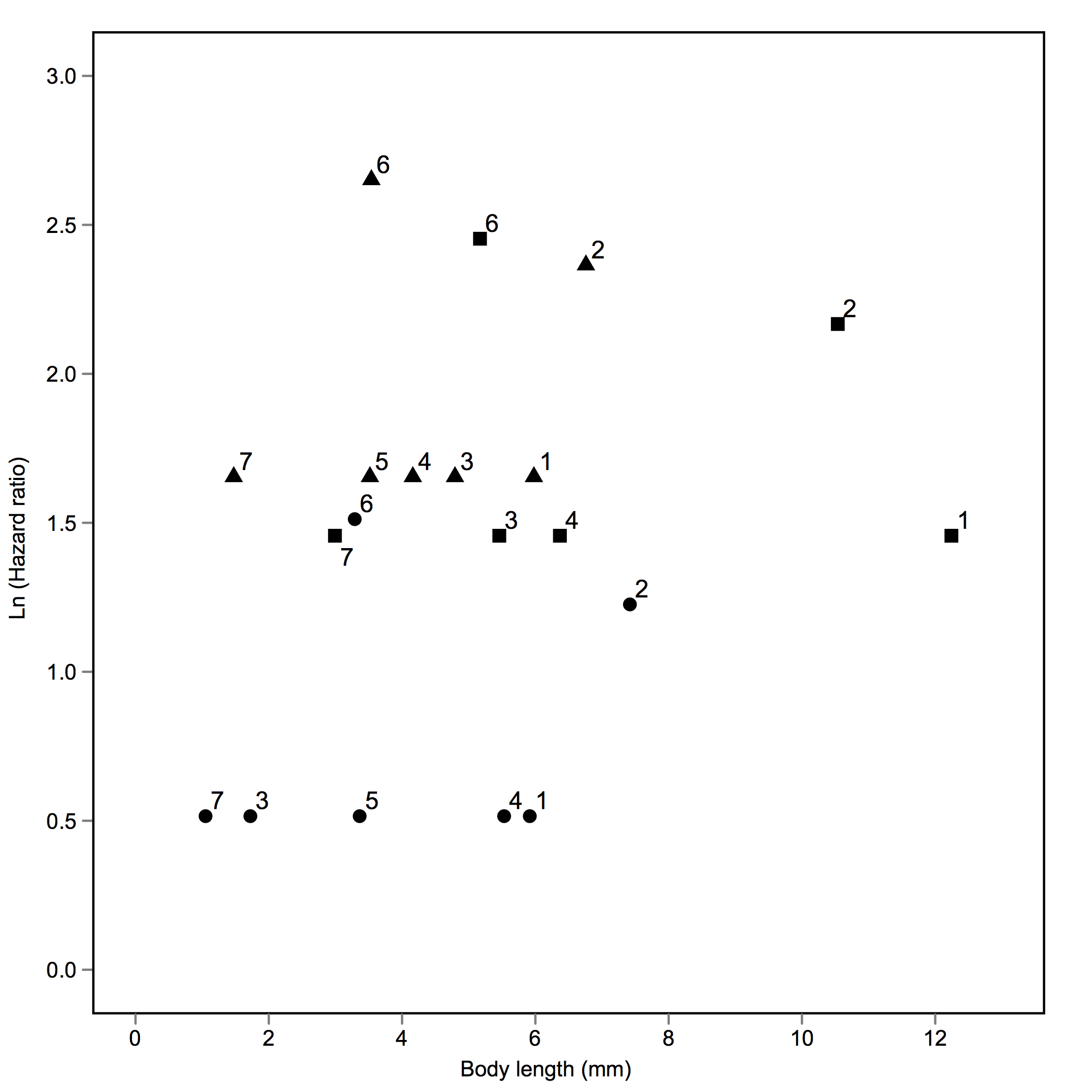

Supplement: Supplementary file 1 — Figure S1. Scatterplot of average body length (mm) against Ln (Hazard ratio) for each ant caste and species. Circles, squares, and triangles represent workers, gynes, and males, respectively. Each number refers to a species: 1 – A. octoarticulatus, 2 – A. rudis, 3 – B. depilis, 4 – C. longipilis, 5 – C. mirabilis, 6 – M. rubra, 7 – O. bauri. [file ece30004-4209-SD1.tif]
