# Supplementary material for: Alate susceptibility in ants
Source: Ecol Evol. 2014 Oct 20;4(22):4209–19. doi: 10.1002/ece3.1291 (PMC4267860; doi:10.1002/ece3.1291)
Supplement: Supplementary file 2 [file ece30004-4209-SD2.doc]

**Supplementary Table 1.** All regression coefficients from the Cox proportional hazards analysis

|  | **Regression coefficient** | **Standard error** | **Lower 95% CI** | **Upper 95% CI** | **P-value** |
| --- | --- | --- | --- | --- | --- |
| *Main effects* | | | | | |
| Fungal | 0.515 | 0.232 | 0.061 | 0.970 | 0.026 |
| Worker | 0.000 |  |  |  |  |
| Gyne | -0.871 | 0.391 | -1.637 | -0.104 | 0.026 |
| Male | 0.979 | 0.240 | 0.508 | 1.450 | < 0.001 |
| *C. mirabilis* | 0.000 |  |  |  |  |
| *C. longipilis* | -0.915 | 0.341 | -1.584 | -0.246 | 0.007 |
| *A. octoarticulatus* | 0.355 | 0.244 | -0.122 | 0.833 | 0.145 |
| *O. bauri* | -1.124 | 0.339 | -1.789 | -0.460 | < 0.001 |
| *M. rubra* | -1.344 | 0.318 | -1.968 | -0.720 | < 0.001 |
| *A. rudis* | -1.538 | 0.307 | -2.140 | -0.937 | < 0.001 |
| *B. depilis* | -2.068 | 0.539 | -3.125 | -1.011 | < 0.001 |
| *Interaction effects* | | | | | |
| Fungus x Gyne | 0.941 | 0.275 | 0.403 | 1.479 | < 0.001 |
| Fungus x Male | 1.139 | 0.219 | 0.711 | 1.567 | < 0.001 |
| Fungus x *C. mirabilis* | 0.000 |  |  |  |  |
| Fungus x *C. longipilis* | 0.711 | 0.364 | 0.002 | 1.419 | 0.049 |
| Fungus x *A. octoarticulatus* | 0.364 | 0.277 | -0.179 | 0.907 | 0.189 |
| Fungus x *O. bauri* | 0.277 | 0.373 | -0.454 | 1.008 | 0.458 |
| Fungus x *M. rubra* | -0.126 | 0.321 | -0.755 | 0.502 | 0.693 |
| Fungus x *A. rudis* | 0.997 | 0.313 | 0.383 | 1.611 | 0.001 |
| Fungus x *B. depilis* | 0.535 | 0.463 | -0.371 | 1.442 | 0.247 |
| Gyne x *C. mirabilis* | 0.000 |  |  |  |  |
| Male x *C. mirabilis* | 0.000 |  |  |  |  |
| Gyne x *C. longipilis* | 0.078 | 0.528 | -0.957 | 1.114 | 0.882 |
| Male x *C. longipilis* | 1.141 | 0.382 | 0.392 | 1.890 | 0.003 |
| Gyne x *A. octoarticulatus* | 0.123 | 0.441 | -0.742 | 0.988 | 0.781 |
| Male x *A. octoarticulatus* | -0.231 | 0.294 | -0.807 | 0.346 | 0.433 |
| Gyne x *O. bauri* | 1.624 | 0.467 | 0.708 | 2.539 | < 0.001 |
| Male x *O. bauri* | 1.159 | 0.471 | 0.235 | 2.082 | 0.014 |
| Male x *M. rubra* | 0.388 | 0.339 | -0.276 | 1.052 | 0.253 |
| Gyne x *A. rudis* | 1.045 | 0.409 | 0.244 | 1.847 | 0.011 |
| Male x *A. rudis* | -0.155 | 0.338 | -0.818 | 0.507 | 0.646 |
| Gyne x *B. depilis* | 1.588 | 0.608 | 0.397 | 2.780 | 0.009 |
| Male x *B. depilis* | 1.490 | 0.555 | 0.402 | 2.578 | 0.007 |

**Supplementary Table 2.** Hazard ratios by species

| **Species** | **Comparison** | | **Hazard ratio** | **Standard error** | **z** | **P-value** |
| --- | --- | --- | --- | --- | --- | --- |
| *C. mirabilis* | ctr-f | ctr-w | 0.419 | 0.391 | -2.226 | 0.034 |
|  | ctr-m | ctr-w | 2.662 | 0.240 | 4.077 | < 0.001 |
|  | fgl-w | ctr-w | 1.674 | 0.232 | 2.224 | 0.034 |
|  | fgl-f | ctr-f | 4.291 | 0.313 | 4.647 | < 0.001 |
|  | fgl-m | ctr-m | 5.230 | 0.240 | 6.903 | < 0.001 |
| *C. longipilis* | ctr-f | ctr-w | 0.419 | 0.391 | -2.226 | 0.034 |
|  | ctr-m | ctr-w | 8.332 | 0.338 | 6.269 | < 0.001 |
|  | fgl-w | ctr-w | 3.408 | 0.3190 | 3.845 | < 0.001 |
|  | fgl-f | ctr-f | 8.734 | 0.381 | 5.688 | < 0.001 |
|  | fgl-m | ctr-m | 10.646 | 0.332 | 7.120 | < 0.001 |
| *A. octoarticulatus* | ctr-f | ctr-w | 0.419 | 0.391 | -2.226 | 0.034 |
|  | ctr-m | ctr-w | 2.662 | 0.240 | 4.077 | < 0.001 |
|  | fgl-w | ctr-w | 1.674 | 0.232 | 2.224 | 0.034 |
|  | fgl-f | ctr-f | 4.291 | 0.313 | 4.647 | < 0.001 |
|  | fgl-m | ctr-m | 5.230 | 0.240 | 6.903 | < 0.001 |
| *O. bauri* | ctr-f | ctr-w | 2.124 | 0.365 | 2.063 | 0.048 |
|  | ctr-m | ctr-w | 8.479 | 0.431 | 4.962 | < 0.001 |
|  | fgl-w | ctr-w | 1.674 | 0.232 | 2.224 | 0.034 |
|  | fgl-f | ctr-f | 4.291 | 0.313 | 4.647 | < 0.001 |
|  | fgl-m | ctr-m | 5.230 | 0.240 | 6.903 | < 0.001 |
| *M. rubra* | ctr-m | ctr-w | 2.662 | 0.240 | 4.077 | < 0.001 |
|  | fgl-w | ctr-w | 1.674 | 0.232 | 2.224 | 0.034 |
|  | fgl-m | ctr-m | 5.230 | 0.240 | 6.903 | < 0.001 |
| *A. rudis* | ctr-f | ctr-w | 1.191 | 0.303 | 0.577 | 0.3378 |
|  | ctr-m | ctr-w | 2.662 | 0.240 | 4.077 | < 0.001 |
|  | fgl-w | ctr-w | 4.537 | 0.263 | 5.762 | < 0.001 |
|  | fgl-f | ctr-f | 11.628 | 0.278 | 8.837 | < 0.001 |
|  | fgl-m | ctr-m | 14.173 | 0.284 | 9.339 | < 0.001 |
| *B. depilis* | ctr-f | ctr-w | 2.050 | 0.541 | 1.326 | 0.166 |
|  | ctr-m | ctr-w | 11.811 | 0.531 | 4.653 | < 0.001 |
|  | fgl-w | ctr-w | 1.674 | 0.232 | 2.224 | 0.034 |
|  | fgl-f | ctr-f | 4.291 | 0.313 | 4.647 | < 0.001 |
|  | fgl-m | ctr-m | 5.230 | 0.240 | 6.903 | < 0.001 |

*ctr- and fgl- represent control and fungus treatment, respectively

*w, f, m represent worker, gyne and male, respectively
